# Periprocedural Use of Oral Anticoagulation Therapy in Patients Undergoing Atrial Fibrillation Ablation

**DOI:** 10.19102/icrm.2018.090801

**Published:** 2018-08-15

**Authors:** Ilir Maraj, Mario D. Gonzalez, Gerald V. Naccarelli

**Affiliations:** ^1^Cardiology Division/EP Section, Department of Medicine, Heart and Vascular Institute, Penn State Health Milton S. Hershey Medical Center, Penn State College of Medicine, Hershey, PA, USA

**Keywords:** Atrial fibrillation, catheter ablation, NOAC

## Abstract

Atrial fibrillation (AF) is the most common sustained arrhythmia encountered in clinical practice today. For those who present with it, one of the most major risks associated with the condition is stroke. AF is associated with a fivefold increased risk of stroke and thromboembolism. Oral anticoagulation has been the cornerstone of stroke prevention in patients with AF. In some individuals who exhibit a higher risk of bleeding, other alternatives for stroke prevention have been sought, including the use of left atrial appendage occlusion devices and surgical exclusion of the left atrial appendage. Catheter ablation is an important treatment strategy in those patients for whom a rhythm control strategy has been selected. This article reviews some of the available anticoagulant drug options and their use prior to, during, and after catheter ablation.

## Introduction

Atrial fibrillation (AF) is the most common sustained arrhythmia encountered in clinical practice. According to current data, more than 33 million people worldwide carry the diagnosis of AF.^1^ In the United States, it is estimated that three to five million people have AF, with the number projected to exceed eight million by 2050.^2^ AF causes many symptoms and contributes to 450,000 hospitalizations and more than 99,000 deaths annually in the US.^3,4^ One of the major risks associated with AF is stroke. AF is associated with a fivefold increased risk of stroke and thromboembolism.^5^ Stroke prevention is therefore essential in patients with AF. Oral anticoagulation has been the cornerstone of stroke prevention in patients with AF, especially in those with a higher risk of stroke. In view of the limitations in patients with higher bleeding risks, other alternatives have been sought for stroke prevention, including the use of left atrial appendage occlusion devices and surgical exclusion of the left atrial appendage. Catheter ablation is an important treatment strategy in cases where a rhythm control strategy has been chosen. In fact, it is the recommended approach in patients with symptomatic paroxysmal and persistent AF with at least one failed antiarrhythmic medication attempt.^6^ Catheter ablation is associated with risks including stroke and transient ischemic attack; therefore, the payment of close attention to anticoagulation before, during, and after the procedure is very important so as to minimize any risks.^7^

## Preprocedural systemic anticoagulation options for stroke prevention

Adequate periprocedural anticoagulation is essential to reduce the risk of stroke, minimize bleeding complications from the ablation procedure, and achieve quality outcomes. Current guidelines recommend administration of an anticoagulant for at least three weeks prior to radiofrequency ablation. Vitamin K antagonists (VKAs) have been the primary anticoagulation medications for patients with a higher risk of stroke (CHA_2_DS_2_-VASc score ≥ 2). With the advent of newer anticoagulation medications, however, the scope of therapy has significantly broadened and these agents have emerged as a viable therapy option in stroke prevention.^8^

### Vitamin K antagonists

Warfarin has been used as an anticoagulant for decades and is the most frequently prescribed medication worldwide.^9^ Warfarin has an inhibitory effect on multiple coagulation factors (II, VII, IX, and X); however, it also has a very narrow therapeutic index and interacts with a variety of foods and medications. In addition, patients need to routinely monitor international normalized ratio (INR) levels and make dose adjustments accordingly.^10^ In the early stages of AF ablation as a procedure, it was standard practice to stop warfarin before the procedure and “bridge” with either enoxaparin or heparin **(Table 1)**. However, this strategy was associated with a high rate of access site bleeding complications.^11^ As a result, multiple studies were performed and now suggest that it is safe to perform AF ablation without interrupting warfarin **(Table 1)**. In a large, multicenter, nonrandomized study in 2010, Di Biase et al. showed that uninterrupted use of warfarin with an INR target > 2 in combination with using an open-irrigated ablation catheter can reduce the risk of periprocedural stroke without increasing the risk of hemorrhagic complications as compared with interrupted warfarin bridged with enoxaparin.^8,12^ The Role of Coumadin in Preventing Thromboembolism in AF Patients Undergoing Catheter Ablation (COMPARE) trial showed that uninterrupted anticoagulation with warfarin was superior in comparison with interrupted warfarin-based strategies employing bridging.^13^ There were more strokes reported in the warfarin-interrupted group as compared with in the warfarin-continued group (INR goal: 2–3.5). Warfarin discontinuation emerged as a strong predictor of periprocedural thromboembolism [odds ratio (OR): 13, 95% confidence interval (CI): 3.1–55.6; p < 0.001].

### Novel oral anticoagulants

Novel oral anticoagulants (NOACs) are relatively new medications approved by the US Food and Drug Administration (FDA) for stroke prevention in patients with nonvalvular AF. The main NOACs approved thus far include dabigatran as a direct thrombin inhibitor and apixaban, rivaroxaban, and edoxaban as factor Xa inhibitors. Evidence and several meta-analyses have demonstrated NOACs to have a similar efficacy and safety to that of warfarin in the setting of catheter ablation.^14^ In addition, when compared with warfarin, NOACs offer more predictable pharmacokinetics, shorter half-lives, and more convenient periprocedural management.

***Dabigatran***. Dabigatran is a direct thrombin inhibitor with selective and reversible binding to thrombin.^15^ The prodrug, dabigatran etexilate, is absorbed and converted to the active metabolite dabigatran.^16^ Almost all of the absorbed prodrug is converted to dabigatran and most of the drug is excreted unchanged in the urine. Peak plasma concentration is reached at approximately two hours after oral administration.^16^ The half-life of dabigatran is 12 hours to 14 hours in elderly healthy subjects.^17^ Dabigatran is contraindicated in instances of severe renal disease. It was approved by the FDA in 2010 for stroke prevention in patients with AF following the release of the results of the Randomized Evaluation of Long-Term Anticoagulation Therapy (RE-LY) trial.^18^ This was a randomized clinical trial that compared two fixed-dose regimens of dabigatran (110 mg twice daily and 150 mg twice daily) with dose-adjusted warfarin in patients with AF and a high risk of stroke. Both dabigatran doses were noninferior to warfarin with respect to stroke prevention. In addition, the 150-mg dose of dabigatran was superior to warfarin with regard to stroke or systemic embolism prevention and the 110-mg dose was superior to warfarin with regard to major bleeding prevention.

Multiple small studies have suggested that minimally interrupted dabigatran therapy is safe and effective. In a study by Kim et al., it was demonstrated that, when dabigatran was withheld for approximately 24 hours before the radiofrequency ablation of AF and resumed four hours after vascular hemostasis, it appeared to be as safe and as effective as the use of uninterrupted warfarin for periprocedural anticoagulation.^19^ However, in a large, multicenter, prospective study, Lakkireddy et al. showed that periprocedural dabigatran use significantly increased the risk of bleeding or thromboembolic complications when compared with uninterrupted warfarin therapy in patients undergoing AF ablation.^20^ In this study, patients taking dabigatran 150 mg orally twice daily prior to ablation were compared with matched patients taking uninterrupted warfarin (therapeutic INR). Dabigatran was withheld about 12 hours prior to ablation.

The Uninterrupted Dabigatran Etexilate in Comparison to Uninterrupted Warfarin in Pulmonary Vein Ablation (RE-CIRCUIT) study was the only randomized study that was a head-to-head comparison of uninterrupted dabigatran (150 mg orally twice daily) versus uninterrupted warfarin (INR: 2–3) for AF abltion.^21^ This was a multicenter study wherein 704 patients were randomized to two groups. The major bleeding incidence in patients in the dabigatran group was significantly lower than that in the warfarin group [five patients (1.6%) versus 22 patients (6.9%); absolute risk difference: 25.3%; relative risk reduction (RR): 77%]. There were six patients who developed cardiac tamponade in the warfarin group versus only one in the dabigatran group. No strokes or thromboembolic events occurred in the dabigatran arm, while one transient ischemic attack occurred in the warfarin arm.

***Rivaroxaban***. Rivaroxaban is an oral, direct, specific factor Xa inhibitor.^22^ It is absorbed rapidly, with maximum plasma concentrations being reached at two hours to four hours after tablet intake. Its oral bioavailability is high (80%–100%) when taken with food and elimination from plasma occurs with a terminal half-life of five hours to nine hours.^23^ The elimination of rivaroxaban occurs via renal elimination of unchanged drug and via metabolic degradation of the drug. Approximately one-third is eliminated in the urine as unchanged active drug.^24^ Multiple studies have shown that rivaroxaban is safe to use in patients with nonvalvular AF undergoing catheter ablation.

Rivaroxaban was approved by the FDA in 2012 for stroke prevention in nonvalvular AF following the results of the Rivaroxaban Once Daily Oral Direct Factor Xa Inhibition Compared with Vitamin K Antagonism for Prevention of Stroke and Embolism Trial in AF (ROCKET AF) study.^25^ The ROCKET-AF trial was a randomized, multicenter trial. In this study, 14,264 patients with nonvalvular AF and at least a moderate risk of stroke (mean CHADS_2_ score, 3.5) were randomized to receive either rivaroxaban (20 mg daily or 15 mg daily if glomerular filtration rate was 30–49 mL/min) or dose-adjusted warfarin (INR: 2–3). At a mean follow-up time of two years, rivaroxaban was found to be noninferior to warfarin for the composite endpoint of stroke or systemic embolism, without increasing the bleeding risk. There were similar rates of thromboembolism (1.1% in the rivaroxaban arm versus 2.1% in the warfarin arm; p = 0.41) and major bleeding (1.6% in the rivaroxaban arm versus 4.2% in the warfarin arm; p = 0.112).

The Study Exploring Two Treatment Strategies in Patients with AF Who Undergo Catheter Ablation Therapy (VENTURE-AF) trial was the first prospective, randomized trial of uninterrupted rivaroxaban and VKAs in patients with nonvalvular AF undergoing catheter ablation.^26^ In this trial, 248 nonvalvular AF patients were assigned to uninterrupted rivaroxaban (20 mg once daily) or to an uninterrupted VKA prior to undergoing catheter ablation and for four weeks afterwards. The primary endpoint was a major bleeding event after catheter ablation. Secondary endpoints included thromboembolic events (composite of stroke, systemic embolism, myocardial infarction, and vascular death) and other bleeding or procedure-attributable events. Notably, it was found that the incidence of major bleeding was low (0.4%; one major bleeding event). Similarly, thromboembolic events were also low (0.8%; one ischemic stroke and one vascular death occurred). All events occurred in the VKA arm and after ablation. This trial demonstrated that the use of uninterrupted rivaroxaban for catheter ablation of AF is feasible and that event rates were similar to those of warfarin.

***Apixaban***. Apixaban is another NOAC recently approved by the FDA in 2012 for stroke prevention in nonvalvular AF patients. It is a direct factor Xa inhibitor that demonstrates rapid absorption and a half-life of 12 hours and is 25% renally excreted.^27^ The results of the Apixaban for Reduction in Stroke and Other Thromboembolic Events in Atrial Fibrillation (ARISTOTLE) trial led to apixaban approval.^28^ In this trial, 18,201 patients with nonvalvular AF and one or more stroke risk factors were randomized to receive either apixaban or warfarin. Specifically, patients were randomized to receive either apixaban 5 mg twice daily (2.5 mg twice daily in patient ≥80 years, body weight ≤ 60 kg, and serum creatinine ≥ 1.5 mg/dL) or dose-adjusted warfarin (INR range: 2–3). With a median follow-up of 1.8 years, apixaban was found to be superior with regard to rate of stroke or systemic embolism (annual incidence: 1.27% versus 1.6%). In addition, apixaban was associated with less major bleeding (2.13% per year versus 3.09% per year). The rate of intracranial hemorrhage was 0.33% per year in the apixaban group and 0.80% per year in the warfarin group (hazard ratio: 0.42, 95% CI: 0.30–0.58; p < 0.001).

In a prospective, multicenter registry of AF patients undergoing radiofrequency catheter ablation, Di Biase et al. demonstrated that uninterrupted apixaban administration was feasible and effective in preventing clinical and silent thromboembolic events without increasing the risk of major bleeding.^29^ In this study, there were no statistical differences regarding major complications (1% versus 0.5%; p = 1), minor complications (3.5% versus 2.5%; p = 0.56), or total bleeding complications (4.5% versus 3%; p = 0.43) between the apixaban and warfarin groups. There were also no symptomatic thromboembolic complications.

The Apixaban Evaluation of Interrupted or Uninterrupted Anticoagulation of AF (AEIOU) trial—a prospective, multicenter clinical study (NCT02608099)—showed that both uninterrupted and minimally interrupted apixaban (one dose withheld) at the time of AF ablation were associated with a very low rate of thromboembolic events and that rates of both major (< 2%) and clinically significant bleeding were similar to those seen with uninterrupted warfarin.^30^

The Apixaban During AF Catheter Ablation: Comparison to Vitamin K Antagonist Therapy (AXAFA) trial (NCT02227550) is another major randomized clinical trial currently underway using uninterrupted apixaban as compared with uninterrupted VKA therapy.

***Edoxaban***. Edoxaban is an oral direct factor Xa inhibitor with 62% oral bioavailability that achieves maximum concentration in one to two hours and is 50% renally excreted.^31,32^ Edoxaban was approved by the FDA in 2015. The major trial that led to edoxaban’s approval was the Effective Anticoagulation with Factor Xa Next Generation in AF—Thrombolysis in Myocardial Infarction 48 (ENGAGE AF-TIMI 48) study.^33^ In this trial, 21,105 patients with moderate- to high-risk AF were randomized to receive dose-adjusted warfarin (INR: 2–3), high-dose edoxaban (60 mg once daily), or low-dose edoxaban (30 mg once daily). With a median follow-up of 2.8 years, both regimens of edoxaban were found to be noninferior to warfarin with respect to the primary efficacy endpoint of stroke or systemic embolism (in the warfarin group, a stroke or systemic embolic event occurred at a rate of 1.5% per year versus 1.18% in patients in the high-dose edoxaban group and 1.61% in the low-dose edoxaban group). The rate of ischemic stroke was similar between high-dose edoxaban and warfarin but was higher with the low-dose edoxaban regimen. The incidence of hemorrhagic stroke and the rate of death from cardiovascular causes were significantly lower with both edoxaban regimens than with warfarin. Edoxaban was also associated with consistently lower dose-related rates of all types of bleeding, including major bleeding, intracranial bleeding, and life-threatening bleeding. The single exception was gastrointestinal bleeding, which occurred more frequently with high-dose edoxaban but less frequently with low-dose edoxaban than it did with warfarin. One interesting finding in this trial was the reduced efficacy of edoxaban in a patient with a glomerular filtration rate > 95 mL/min.^33^ In a recent study evaluating patients in the ENGAGE AF-TIMI 48 trial undergoing AF ablation, treatment with edoxaban was associated with a low risk of ischemic and bleeding events during the first 30 days after ablation.^34^

Overall, NOACs compare favorably to warfarin in the prevention of stroke in patients with nonvalvular AF. A recent large meta-analysis that included 71,683 patients from four major trials comparing NOACs versus warfarin for stroke prevention (ie, RE-LY, ARISTOTLE, ROCKET AF, and ENGAGE AF-TIMI 48)^35^ showed that NOACs had a favorable risk–benefit profile, with significant reductions in stroke, intracranial hemorrhage, and mortality and with similar major bleeding rates as warfarin but increased gastrointestinal bleeding. NOACs reduced stroke or systemic embolic events by 19% as compared with warfarin (RR: 0.81, 95% CI: 0.73–0.91; p < 0.0001), mainly driven by a reduction in hemorrhagic stroke (RR: 0.49, 95% CI: 0.38–0.64; p < 0.0001). NOAC use also significantly reduced all-cause mortality (RR: 0.90, 95% CI: 0.85–0.95; p = 0.0003) and intracranial hemorrhage (RR: 0.48, 95% CI: 0.39–0.59; p < 0.0001) but increased gastrointestinal bleeding (RR: 1.25; 95% CI: 1.01–1.55; p = 0.04).

## Intraprocedural anticoagulation

Systemic anticoagulation with unfractionated heparin during AF ablation is very important. It is recommended that heparin be administered prior to or immediately following transseptal puncture and adjusted to maintain a target activated clotting time (ACT) of 300 seconds or more.^7^ It has been shown that early anticoagulation with heparin significantly decreases the risk of clot formation.^36,37^

In a recent large meta-analysis of 7,150 patients, Briceno et al. demonstrated that performing AF catheter ablation with a target ACT of > 300 seconds decreased the risk of thromboembolic events without increasing bleeding risk.^38^ Patients with ACTs of > 300 seconds had fewer thromboembolic events (OR: 0.51, 95% CI: 0.35–0.74) and less bleeding (OR: 0.70, 95% CI: 0.60–0.83) than did patients with ACTs of < 300 seconds when using any type of oral anticoagulation. Another interesting finding in this study was that the use of VKAs was associated with reduced heparin requirements and less time needed to achieve a target ACT as compared with NOAC use.

Most operators administer heparin just prior to transseptal puncture. According to the 2017 Heart Rhythm Society/European Heart Rhythm Association/European Cardiac Arrhythmia Society/Asia Pacific Heart Rhythm Society/Sociedad Latinoamericana de Estimulacion Cardiaca y Electrofisioexpert consensus statement on catheter and surgical ablation of AF, the heparin loading dose should be administered followed by heparin infusion. The ACT level should be checked initially every 10 minutes to 15 minutes until therapeutic levels are achieved and then every 15 minutes to 30 minutes thereafter. The initial heparin loading dose should be 50 units/kg in patients anticoagulated with warfarin, 75 units/kg in patients not on anticoagulation, and 120 units/kg in patients on NOACs. The heparin dose should be adjusted to maintain an ACT of 300 seconds to 350 seconds throughout the procedure.^7^ Transseptal sheaths should be infused continuously with heparinized saline to reduce the risk of thrombus formation.^36^

## Anticoagulation following atrial fibrillation ablation

Due to postablation hypercoagulability, it is a consensus that anticoagulation should be continued for at least two months after ablation, irrespective of stroke risk or rhythm status.^7^ Immediately postprocedure, if a patient was on a NOAC, most operators would restart the medication three hours to five hours after sheath removal. If a patient was on warfarin and the procedure was performed on therapeutic INR (uninterrupted strategy), a warfarin dose can be given at the usual time of night. If considering a patient undergoing AF ablation who is utilizing the interrupted coagulation strategy, then low-molecular-weight heparin, enoxaparin 0.5 mg/kg to 1 mg/kg twice daily, or heparin can be given until the INR becomes therapeutic.^7,39^ In patients with a high stroke risk profile (CHA_2_DS_2_-VASc score ≥ 2), current guidelines recommend long-term anticoagulation after AF catheter ablation.

## Anticoagulation reversal agents

To reduce the severity of bleeding complications from anticoagulation, reversal agents are frequently required. The most common reversal agents to treat warfarin toxicity are vitamin K and fresh frozen plasma (FFP). The lack of antidotes and reversal agents for NOACs (with the exception of dabigatran) quite often limits the use of these medications.

### Warfarin antagonists

Vitamin K is the essential cofactor for the synthesis of vitamin K–dependent proteins and serves as the first-line treatment for patients with warfarin-associated coagulopathy.^40^ FFP contains coagulation factors—including factors II, VII, IX, and X in concentrated form—and is used for combating severe bleeding due to warfarin toxicity.^40,41^

Four-factor prothrombin complex concentrate (4F-PCC) **(Table 2)** contains factors II, VII, IX, and X prepared from plasma and was approved by the FDA in 2013 for use in cases of severe bleeding due to VKA therapy. 4F-PCC products are lyophilized items that are administered in smaller volumes over shorter periods of time.^42^

### Novel oral anticoagulant antagonists

Idarucizumab is a monoclonal antibody fragment that binds dabigatran with high affinity and was approved by the FDA in 2015 following the completion of the Reversal Effects of Idarucizumab on Active Dabigatran (RE-VERSE AD) trial.^43^ In this trial, patients received 5 g of intravenous idarucizumab **(Table 2)**. This dose was given as two 50-mL bolus infusions, each containing 2.5 g of idarucizumab, administered no more than 15 minutes apart. The 5-g dose was calculated to reverse the total body load of dabigatran that was associated with the 99^th^ percentile of the dabigatran levels.

Andexanet alfa (andexanet) is a specific reversal agent that is designed to neutralize the anticoagulant effects of both direct and indirect factor Xa inhibitors.^44^ The ongoing Prospective, Open-label Study of Andexanet Alfa in Patients Receiving a Factor Xa Inhibitor Who Have Acute Major Bleeding (ANNEXA-4) phase IIIb/IV study (NCT02329327) is aimed at evaluating the efficacy and safety of andexanet in patients with factor Xa inhibitor–associated acute major bleeding.

Several coagulation factor products have been investigated in small studies and are available for emergent periprocedural bleeding reversal in patients on NOACs. PCCs, recombinant factor VIIa (rFVIIa), and factor eight inhibitor bypassing activity (FEIBA) **(Table 2)** are already available for off-label use. PCCs have been shown to reverse the anticoagulation effects of factor Xa in healthy volunteers,^45^ animal models,^46^ and in vitro studies of healthy donor blood.^47^ rFVIIa has been shown to improve coagulation parameters in small studies using healthy volunteers on dabigatran.^48^ It similarly resulted in improvements in laboratory parameters in studies of factor Xa inhibitors using animal models and healthy donor blood.^46,47^ In an animal study, rFVIIa and PCC partially improved laboratory parameters; however, they did not reverse rivaroxaban-induced bleeding.^46^ In addition, there are studies that suggest that rFVIIa use significantly increased the risk of arterial thromboembolic events as compared with a placebo, an effect that was more pronounced in the elderly population.^49^ FEIBA contains the proenzymes of the prothrombin complex factors, prothrombin, FVII, FIX, and FX, but only very small amounts of their activation products, with the exception of FVIIa, which is contained in FEIBA in greater amounts.^50^ Small studies have shown improvement in coagulation parameters with FEIBA for factor Xa inhibitors as well as for dabigatran.^48^ The results were not consistent across all studies and coagulation parameters. Based on the limited data available, 4F-PCC is a reasonable option for emergency reversal of severe life-threatening bleeding in patients anticoagulated with oral factor Xa inhibitors.^51^

## Conclusions

As outlined above, therapeutic anticoagulation is the mainstay of safely performing AF ablation procedures to minimize thromboembolic events. Stroke remains one of the most serious complications of AF ablation but, at the same time, there is a concern regarding intraprocedural and postprocedural bleeding stemming from the use of anticoagulants during this procedure. It should be remembered that anticoagulation is not the only factor responsible for bleeding or embolic phenomena in patients undergoing catheter ablation of AF. Transseptal puncture, high-caliber contact force, and/or induction of a “pop lesion” due to high tissue temperatures during radiofrequency energy delivery can account for cardiac perforation and tamponade irrespective of the anticoagulant used. On the other hand, char formation, endocardial damage, and thrombus formation due to insufficient heparin administration may contribute to an increased risk of stroke.^52^

New data from NOAC trials suggest that these agents can be used safely in an uninterrupted fashion, although they have an effect on the amount of heparin required to achieve a therapeutic ACT. The major concern of periprocedural uninterrupted use of NOACs has been the lack of reversal agents available. As mentioned before, idarucizumab is the only agent available that can reverse the effect of dabigatran, while agents for some other NOACs remain nonestablished. Cardiac tamponade during catheter ablation needs to be managed with pericardial drainage and hemodynamic support without waiting for a reversal agent. In addition, the administration of coagulation products (eg, PCCs, rFVIIa, FEIBA) appears to confer a protective action against periprocedural bleeding; notably, this is the recommended approach until more reversal agents become available.^7,51,53^ The development of NOAC reversal agents may help to minimize concerns regarding the use of NOACs for these procedures. Until more data are available, high-risk AF patients should be kept on long-term oral anticoagulation therapy after AF ablation.

## Figures and Tables

**Table 1:** Strategies to Manage Anticoagulation Before, During, and After Ablation

Strategy	Before Ablation		During Ablation		After Ablation	
Interrupted warfarin	•	Five days before the procedure, stop warfarin and bridge with LMWH	•	Administer heparin	•	Stop heparin
			•	Achieve an ACT > 300 seconds	•	Consider protamine
					•	Remove sheath when ACT is less than 200–250 seconds^7^
					•	Restart warfarin and bridge with LMWH until INR is in therapeutic range
Interrupted NOAC	•	Five days before the procedure, stop NOAC and switch to warfarin or bridge with LMWH	•	Administer heparin	•	Stop heparin
			•	Achieve an ACT > 300 seconds	•	Consider protamine
					•	Remove sheaths when ACT is less than 200–250 seconds^7^
					•	Resume NOAC three to five hours after sheath removal
Uninterrupted warfarin	•	Continue warfarin	•	Administer heparin	•	Stop heparin
			•	Achieve an ACT > 300 seconds	•	Consider protamine
					•	Remove sheath when ACT is less than 200–250 seconds^7^
					•	Continue warfarin
Uninterrupted or minimally interrupted NOAC	•	Stop NOAC 12–24 hours before the procedure or continue uninterrupted	•	Administer heparin	•	Stop heparin
			•	Achieve an ACT > 300 seconds	•	Consider protamine
					•	Remove sheath when ACT is less than 200–250 seconds^7^
					•	Resume NOAC use three to five hours after sheath removal

LMWH: low-molecular-weight heparin; ACT: activated clotting time; INR: international normalized ratio; NOAC: novel oral anticoagulant.

**Table 2:** Periprocedural Bleeding Reversal Agents

Warfarin		Direct Thrombin Inhibitors		Factor Xa Inhibitors	
Intravenous 4F-PPC^51^		Intravenous idarucizumab 5 g		Intravenous 4F-PPC^51^	
•	INR: 2–4, 25 units/kg	•	Give two 50-mL bolus infusions, each containing 2.5 g of idarucizumab, no more than 15 minutes apart^43^	•	50 units/kg
•	INR: 4–6, 35 units/kg				
•	INR: > 6, 50 units/kg				
If 4F-PPC is not available, use 10–15 mL/kg of plasma		If idarucizumab is not available, use 50 units/kg of 4F-PPC^51^		Other coagulation products include FEIBA, rFVIIa, aPCC	
		Other coagulation products include FEIBA, rFVIIa, aPCC			

4F-PPC: four-factor prothrombin complex concentrate; INR: international normalized ratio; FEIBA: factor eight inhibitor bypassing activity; rFVIIa: recombinant factor VIIa; aPCC: activated prothrombin complex concentrate.
